# Transcriptome-Guided Insights Into Plastic Degradation by the Marine Bacterium

**DOI:** 10.3389/fmicb.2021.751571

**Published:** 2021-09-27

**Authors:** Alka Kumari, Nasreen Bano, Sumit Kumar Bag, Doongar R. Chaudhary, Bhavanath Jha

**Affiliations:** ^1^Plant Omics Division, CSIR-Central Salt and Marine Chemical Research Institute, Bhavnagar, India; ^2^Academy of Scientific and Innovative Research (AcSIR), CSIR, Ghaziabad, India; ^3^Molecular Biology and Biotechnology, CSIR-National Botanical Research Institute, Lucknow, India

**Keywords:** biodegradation, polyethylene terephthalate, marine bacteria, genome, transcriptome

## Abstract

Polyethylene terephthalate (PET) is a common single-use plastic that accumulated in the environment because of its non-degradable characteristics. In recent years, microbes from different environments were found to degrade plastics and suggested their capability to degrade plastics under varying environmental conditions. However, complete degradation of plastics is still a void for large-scale implications using microbes because of the lack of knowledge about genes and pathways intricate in the biodegradation process. In the present study, the growth and adherence of marine *Bacillus* species AIIW2 on PET surface instigating structural deterioration were confirmed through weight loss and hydrophobicity reduction, as well as analyzing the change in bond indexes. The genome-wide comparative transcriptomic analysis of strain AIIW2 was completed to reveal the genes during PET utilization. The expression level of mRNA in the strain AIIW2 was indexed based on the log-fold change between the presence and absence of PET in the culture medium. The genes represent carbon metabolism, and the cell transport system was up-regulated in cells growing with PET, whereas sporulation genes expressed highly in the absence of PET. This indicates that the strain AIIW2 hydrolyzes PET and assimilated via cellular carbon metabolism. A protein–protein interaction network was built to obtain the interaction between genes during PET utilization. The genes traced to degrade PET were confirmed by detecting the hydrolytic product of PET, and genes were cloned to improve PET utilization by microbial system as an eco-friendly solution.

## Introduction

Polyethylene terephthalate (PET) is the most common single-use synthetic polymer, primarily used in the packaging industries such as plastic bags, bottles, and films (Andrady and Neal, 2009). Within the decades of its discovery, it has steered its spread in different ecosystems (Lithner et al., 2011; Wang et al., 2020). The need for remediation approaches is particularly urgent to combat non-degradable synthetic polymer accumulation. The biological approaches to remediate plastic wastes could provide an eco-friendly solution. The growing concerns for an efficient system meant for biodegradation of plastics are mainly due to the limitations in the conventional plastic disposal methods that cause the release of harmful chemicals in the surrounding environment that affect the life of living organisms at various trophic levels (Tokiwa and Calabia, 2004; Hermanová et al., 2015).

In recent years, microorganisms or their enzymes are increasingly reported for degradation and decomposition of plastics. During PET degradation study, a thermophilic hydrolase from *Thermobifida fusca* had combined characteristics of lipase and esterase (Mueller, 2006). An esterase from polyester-degrading bacterium (*Thermobifida halotolerans*) was cloned in *Escherichia coli* found to hydrolyze PET and *bis*-(benzoyloxyethyl) terephthalate into terephthalic acid and mono-(2-hydroxyethyl) terephthalate (Ribitsch et al., 2012). The two-novel esterase from anaerobic *Clostridium botulinum* strain (ATCC 3502) were found to hydrolyze polyester, which was cloned in *E. coli* BL21 (Perz et al., 2016). The engineered *Comamonas testosteroni* strain can degrade PET particles under alkaline conditions (Gong et al., 2018). Subsequently, a bacterium *Ideonella sakaiensis* could utilize PET as carbon and degrade by secretion of PETase (Austin et al., 2018). The aliphatic, aromatics, polyaromatic hydrocarbon, phthalate, polyethylene, and polyisoprene degradation genes were reported in the *Rhodococcus* (Zampolli et al., 2019). Janczak et al. (2020) demonstrated the ability of rhizospheric bacterial strain (*Serratia plymuthica*) for degradation of PET in cultivated and compost soil, as well as the presence of 155 genes for xenobiotics biodegradation in the genome.

In marine environments, plastic-degrading microorganisms are often found on waste plastic surfaces. A recent study of the taxonomic pattern of microbes associated with plastics from the marine environment showed that the recurring groups and families of Erythrobacteraceae and Rhodobacteraceae (Alphaproteobacteria) bacteria, Flavobacteriaceae (Bacteriodetes), and Cyanobacteria (*Phormidium*) were documented (Roager and Sonnenschein, 2019). A carboxylic ester hydrolase from the marine *Pseudomonas aestusnigri* was identified to degrade PET, which had amino acid sequence homology with type IIa family of PET hydrolytase (Bollinger et al., 2020). Sarkhel et al. (2020) reported marine bacterium and fungus, which degraded 35 and 22% plastic waste stripes, respectively, within 6 weeks. PETase had been displayed on the yeast surface (*Pichia pastoris*) to develop the whole-cell biocatalysis for improved PET degradation efficiency at higher pH and temperature stability (Chen et al., 2020). Notably, microorganisms have a great potential to degrade plastics, which could be amplified to a higher level through an understanding of underlying pathways, ultimately to come up with novel bioremediation approaches.

The functional analysis of available genome data could provide invaluable information for developing and designing strategies to attenuate plastic non-degradability (Zampolli et al., 2019). The metabolic networks with the catabolic enzymes could be exploited from genomics and transcriptomic networking in bioremediation applications.

This study aimed to resolve the PET mineralization process by the marine *Bacillus* strain AIIW2. We used a comparative transcriptomic approach to trace genes involved in plastic degradation that will highlight potential bottlenecks in the microbial PET mineralization process, which could become preferential targets for optimizing PET degradation by environmental microorganisms. The fundamental idea of plastic degradation now shows a two-step process: the hydrolysis of polymer into shorter fragments followed by mineralization by the candidate microorganism.

## Materials and Methods

### Polyethylene Terephthalate

The bacterial degradation was measured in commercially available standard PET film (Sigma-Aldrich, United States). The approximate average molecular weight of PET film was 19,500 g mol^– 1^ and density 1.38 g cm^– 3^ used in the present study.

### Bacterial Strain and Growth Conditions

The *Bacillus* species AIIW2 (KU877334) was initially isolated from plastic waste collected from the marine environment (Kumari et al., 2019). The strain AIIW2 used in this study was previously found to degrade different plastics through extensive analytical and microscopic studies (Kumari et al., 2019). The bacterial strain was cultured and maintained in Zobell marine broth at 30°C and 120 revolutions/min (rpm) or on solid Zobell marine agar plates with 1.8% agar. For bacterial degradation study, 1 mg mL^– 1^ of PET film was incubated in 30 mL of Bushnell and Haas broth (BHB) inoculated with the *Bacillus* species AIIW2, and another flask without any carbon source was taken as control incubated at 30°C under shaking condition. Before inoculation, PET film was washed with sterilized MilliQ water thrice, dried in laminar air flow, and UV-irradiated for sterilization.

### Scanning Electron Microscopy Study

The morphological change on the surface of PET film due to bacterial activity was assessed using a field emission scanning electron microscope (FE-SEM, JSM-7100F, Jeol Ltd., United States). The bacterial-treated and -untreated PET films were taken out after 30 days of incubation and fixed with 2% glutaraldehyde (2 h), subsequently dehydrated with 30, 50, 70, and 100% ethanol for 30 min each, and then vacuum-dried in a desiccator. Vacuum dried films were coated with gold before scanning.

### Weight Loss Assay

The bacterial degradation of PET film was evaluated through the dry weight reduction method described earlier (Kumari et al., 2019). Briefly, 1 mg mL^– 1^ of preweighted PET film were extracted from the bacterial culture maintained at 30°C under shaking condition in BHB medium after every 15 days up to 90 days to measure the degradation. The extracted PET films were washed with 2% sodium dodecyl sulfate (SDS) to remove attached cells and rinsed with MilliQ water thrice. The washed PET films were dried overnight at 50°C to determine the dry weight. The biodegradation efficiency of the marine bacterium was determined through a weight loss of the PET films when offered as a sole source of carbon.

### Carbon Mineralization of Polyethylene Terephthalate

The PET mineralization into carbon dioxide resulting from hydrolysis was quantified through the titrimetric method (ISO 14855, 2005; Mohee et al., 2008; Funabashi et al., 2009). The *Bacillus* species AIIW2 was grown in 300 mL of BHB medium supplemented with 1 mg mL^– 1^ of PET film as a carbon source. Carbon mineralization was also studied in a bacterial culture grown without PET in BHB medium considered as a control treatment. The inoculated and control flasks were connected with 20 mL of sterilized 0.1 N sodium hydroxide through silicon tubing. The sodium hydroxide flasks were changed every 5 days of incubation, and CO_2_ production was measured in the initial flask through titration against 0.1 N HCl up to 35 days of incubation at 30°C (ISO 14855, 2005; Mohee et al., 2008). The CO_2_ evolution during the remineralization of PET film was calculated from the CO_2_ evolved from the control flask and the initial carbon content of PET films.

### Hydrophilicity Measurement of Plastic Films

Water contact angles of the PET films after incubating with bacterial strain AIIW2 were measured using a Drop Shape Analysis System DSA 100 (KRÜSS GmbH, Hamburg, Germany). The PET films were removed from the culture medium and washed with 2% SDS followed by rinsing with distilled water and oven-dried overnight at 50°C. The PET films were analyzed every 15 days of incubation up to 90 days by dropping water on the surface, and the contact angle was measured at three points in triplication (Ribitsch et al., 2012).

### Bond Indexes

Structural changes were analyzed in PET films (1 mg mL^– 1^) incubated in the bacterial culture and an uninoculated control medium every 30 days after incubation for 90 days through Fourier transform infrared spectroscopy (FTIR) (Spectrum EX, PerkinElmer) in the frequency range of 400–4,000 cm^– 1^ with a resolution of 1 cm^– 1^. The relative absorbance intensities of the ester carbonyl bond were evaluated using the following formula (Albertsson et al., 1987):

Keto Carbonyl Bond Index (KCBI) = *I*_1715_/*I*_1465;_ Ester Carbonyl Bond Index (ECBI) = *I*_1740_/*I*_1465_;

Vinyl Bond Index (VBI) = *I*_1650_/*I*_1465_; Internal Double Bond Index (IDBI) = *I*_908_/*I*_1465_.

### RNA Extraction and Sequencing

The *Bacillus* AIIW2 was incubated for 7 days at 30°C in a shaker incubator by supplementing with PET as treatment and without PET as control experiment in BHB medium; RNA was extracted using the RNeasy Mini Kit (Qiagen, Germany) according to the manufacturer’s instructions. The flasks were maintained in triplicates and were prepared for RNA extractions. The mRNA transcripts were sequenced as forward and reversed read files. The fastq sequence file with overlapping paired-end reads was created (MedGenome Labs Pvt., Ltd.). Illumina MiSeq 2 × 100-bp paired-end libraries with multiplex adaptors were prepared with an internal PhiX control by the Genoscreen platform (Malausa et al., 2011).

### Sequencing Data Filtration and Differential Expression Analysis

The raw reads from sequencing data of all the replicates were processed for the quality check using FastQC (version 0.11.2) (Andrews, 2010). The low-quality reads were removed through a quality filter from the fastq raw reads using NGS QC toolkit v2.3.3 with stringent filtering criteria (Patel and Jain, 2012). The reads with *Q* < 30 bases were removed. Cufflink (version 2.2.1) was used to assemble the reads into transcripts on the basis of the mapping results (Trapnell et al., 2012). Filtered reads were aligned with the genome using bowtie2 (version 2.3.5.1) (Langmead and Salzberg, 2012) and Cufflinks. For evaluation of gene expression levels, the FPKM (per kilobase of exon per million fragments mapped) method was used. The analysis of differential expression was carried out using the cuffdiff (Trapnell et al., 2012). Significant differentially expressed genes (DEGs) were shown through heat map using an R script, and the scale of the heat map was set according to data values.

### Functional Characterization

Gene ontology (GO) of DEGs was applied to study the gene functions. The GO annotation was performed using ShinyGO v0.61 (Ge et al., 2020) and illustrated through the R studio. The GO annotation was retrieved using a singular enrichment analysis statistical test at *p* < 0.05.

### Pathway Analysis

To retrieve the KO [kyoto encyclopedia of genes and genomes (KEGG) Orthology] identifiers from KAAS database,^1^ entire assembled sequences were utilized (Moriya et al., 2007). These KEGG identifiers were used in KEGG^2^ to retrieve all possible metabolic pathways (Kanehisa et al., 2016).

### Quantitative Real-Time Polymerase Chain Reaction

The expression profile of genes from RNA-seq analysis was validated through reverse transcriptase–polymerase chain reaction (RT-PCR). The total RNA was isolated from *Bacillus* species AIIW2 which were cultured in BHB supplemented with and without PET for 7 days. Reverse transcription was performed with QuantiTect Reverse Transcription Kit (Qiagen) to obtain the corresponding cDNA library according to the manufacturer’s instruction. Thirteen up-selected and 12 down-selected genes were validated by the RNA-seq differential gene expression data via RT-PCR. Amplification was performed in 20-μL volume that contained 1 μL of the reverse-transcribed RNA samples, 10 μL of SYBR Green Master Mix (Qiagen) and 20 mM of each primer (Supplementary Table 1). Thermocycling conditions were as follows: 30 s at 95°C, followed by 40 cycles of 5 s at 95°C, 10 s at 58°C and 45 s at 72°C, and one cycle of 15 s at 95°C, 1 min at 60°C and 15 s at 60°C for 35 cycles of amplification in Real-Time PCR Detection System (Bio-Rad). Expression of the housekeeping gene, 16S rRNA was used as a reference gene to normalize tested genes in *Bacillus* species AIIW2. The Ct value calculated from 16S rRNA reference gene was used to determine the relative abundance of target transcripts (Su et al., 2016). The RT-PCR results were compared with the transcriptome data to detect the correlation of each gene expression.

### Reverse-Phase High-Pressure Liquid Chromatography

The product of PET hydrolysis was determined through reverse-phase high-pressure liquid chromatography (HPLC, RID-10A, Shimadzu, Japan). The bacterial strain AIIW2 was cultured for 30 days in 100 mL of BHB supplemented with PET film as a carbon source at 30°C under shaking conditions. After incubation, the culture supernatant was centrifuged at 10,000 rpm for 10 min and freeze-dried. The freeze-dried culture supernatant was acidified up to pH 2.5 using 2 M HCl, and hydrolysis products were extracted with ethyl acetate. The extracted hydrolyzed products were filtered with 0.2-μm filter and 20 μL injected in C_18_ column (Shimadzu, Japan). The mobile phase was 20% acetonitrile, 20% 10 mM phosphoric acid, and 60% (vol/vol) MilliQ water with a flow rate of 1 mL min^– 1^ at wavelength 241 nm. The peaks were quantified through standard curves of terephthalate (TPA), mono-(2-hydroxyethyl) terephthalic acid (MHET), and *bis*(2-hydroxyethyl) terephthalate (BHET) (concentration range, 0.1–1 mM).

### String Network

Carboxylesterase and aldehyde dehydrogenase genes were analyzed for their interaction network in STRING database^3^ (Szklarczyk et al., 2011) using the Cytoscape 2.8 version (Smoot et al., 2011).

## Results

### Polyethylene Terephthalate Degradation and Remineralization

**FIGURE 1:**
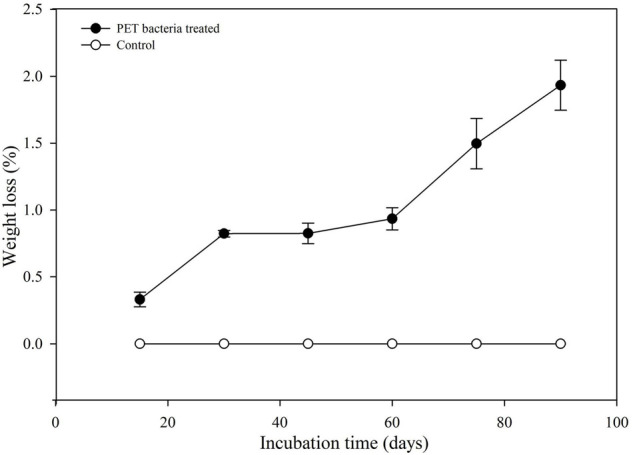
Weight loss of PET film after incubation with *Bacillus* species AIIW2.

To determine the degradation of standard PET film by marine *Bacillus* species AIIW2, weight reduction of PET film was observed every 15 days up to 90 days of the incubation period with the bacteria. The weight loss of PET film in percent was observed as 0.42, 0.82, 0.83, 0.94, 1.27, and 1.93% degradation after 15, 30, 45, 60, 75, and 90 days of incubation, respectively (Figure 1). However, no weight reduction was observed in PET film incubated without bacteria.

**FIGURE 2:**
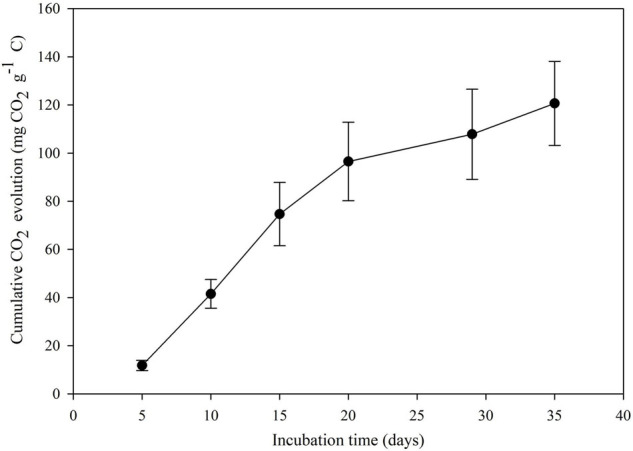
Cumulative CO_2_ evolution by bacterial strain during growth with PET.

The CO_2_ evolution was estimated to confirm PET assimilation and mineralization by bacterial strain AIIW2. It was observed that the conversion of PET into CO_2_ (cumulative) was 11.78, 41.50, 74.29, 96.16, 116.49, and 128.07 mg CO_2_ g^– 1^ of C from days 5, 10, 15, 20, 30, and 35, respectively (Figure 2). The experiment was compared against control in which bacterial cultures were maintained without any carbon source.

### Bacteria Adherence and Morphological Disruption

**FIGURE 3:**
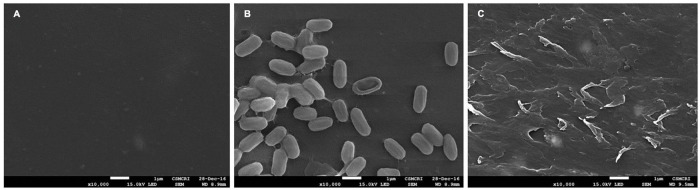
SEM image of PET film after 30 days of incubation **(A)** control film, **(B)** with *Bacillus* species AIIW2, and **(C)** PET surface after washing off the bacterial cells.

The colonization of the bacteria cells on the PET surface was confirmed through SEM after 30 days of incubation, and it was observed that the bacterial strain AIIW2 colonized and disrupted the PET surface, whereas untreated control film remains intact (Figure 3).

### Hydrophilicity Measurement of Polyethylene Terephthalate Films

**FIGURE 4:**
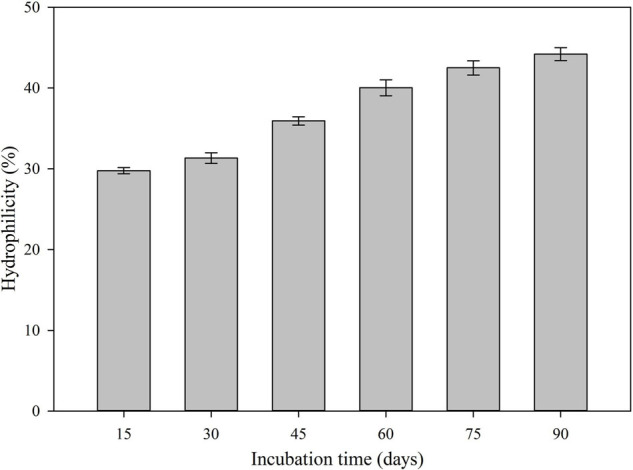
The hydrophilicity of bacterial treated PET surface during the incubation period.

An analysis of the water contact angle on the PET film showed that after 90 days of bacterial treatment with strain AIIW2 was reduced from 77.3° (control) to 55.8° (bacteria treated) (Supplementary Figure 1). The results also indicated that the inoculation of bacterial strain AIIW2 decreased the hydrophobicity of the PET film and increased the surface hydrophilicity from 29.7 to 44.2% in 90 days (Figure 4). As the PET surface became less hydrophobic, it will be less resistant to subsequent degradation by the bacterial cells.

### Fourier Transform Infrared Spectroscopy

**FIGURE 5:**
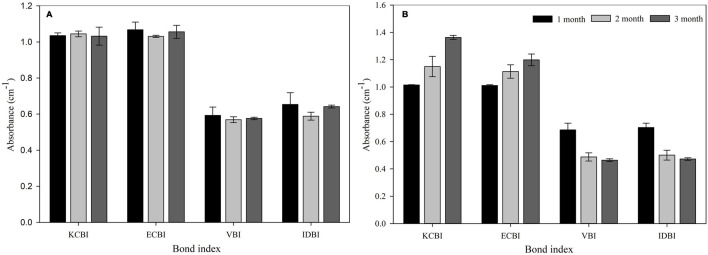
Bond indexes of PET film after incubation **(A)** without and **(B)** with *Bacillus* species AIIW2 for 3 months. KCBI, Keto Carbonyl Bond Index; ECBI, Ester Carbonyl Bond Index; VBI, Vinyl Bond Index; IDBI, Internal Double Bond Index.

Formation or disappearance of acids (1,715 cm^– 1^), ketones (1,740 cm^– 1^), and double bonds (1,640 and 908 cm^– 1^) was monitored using the FTIR to determine the mechanism of the biodegradation process. KCBI, ECBI, VBI, and IDBI were calculated and confirmed the structural transformation of PET due to bacterial action. The bond indexed was observed to be unchanged in PET during the 90 days of incubation period in BHB medium without bacteria. However, bacterial treated PET film had increased KCBI and ECBI and a reduction in VBI and IDBI over the incubation period of 90 days (Figure 5).

### Read Quality Filtration

Misassembled transcripts from RNA-seq were filtered out with the help of genome sequence data of *Bacillus* species AIIW2 (KY694465) (Kumari et al., 2020). A total of 63,27,406 and 56,70,745 high-quality raw reads were generated out of three replicates of transcriptome sequencing data of control and treated samples, respectively. After filtration, there were a total of 63,26,176 and 56,69,934 high-quality filtered reads were retrieved in control and treated samples, respectively. The detailed description of filtered reads is given in Supplementary Table 2. The Short Read Achieve (SRA) data were deposited in NCBI databank with SRA identifier SRP279031 and accession number MZ322848.

### Identification of Differentially Expressed Genes

**FIGURE 6:**
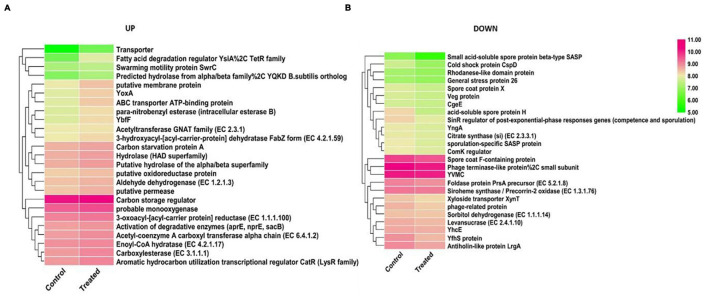
The expression patterns of the DEGs identified between control and treated samples. The heat map represents the relative expression levels of 25 genes examined in up-regulated **(A)** and down-regulated **(B)** genes based on log2 FPKM values.

The RNA-seq profiling of *Bacillus* species AIIW2 while growing with PET as carbon source and without PET was performed to unveil the genes intricate in the degradation process. The differential gene expression of bacterial culture when grown with PET was compared with that grown without PET. A total of 3,992 genes were generated via mapping of reads to the reference genome. The comprehensive information of genes and their annotations are given in Supplementary Table 3. Identification of differential expression of genes during PET utilization was based on FPKM calculation of each gene, and a total of 2,031 DEGs were detected, among which 1,073 genes were up-regulated and 958 genes were down-regulated (Supplementary Table 4). Top up-regulated and down-regulated genes are shown in Figure 6 through heat map using the log2 FPKM values. Based on analysis of expression of these genes, enoyl-CoA hydratase (EC 4.2.1.17), acetyltransferase family (EC 2.3.1), activation of degradative enzymes, aldehyde dehydrogenase (EC 1.2.1.3), 3-oxoacyl-[acyl-carrier protein] reductase (EC 1.1.1.100), hydrolases, carboxylesterases, and putative permeases were up-regulated in bacteria-treated PET as carbon source genes. Likewise the expression of small acid-soluble spore protein beta-type SASP, acid-soluble spore protein H, cold shock protein CspD, YfhS protein, SinR regulator (post–exponential-phase responses, i.e., competence and sporulation) genes, antiholin-like protein LrgA, and sporulation-specific SASP protein were down-regulated genes. The 28.11% of hypothetical genes differentially also expressed when bacteria were grown with PET, indicating that the proteins with unknown functions were involved directly or indirectly for substrate adaptation.

### Functional Analysis of Differentially Expressed Genes

**FIGURE 7:**
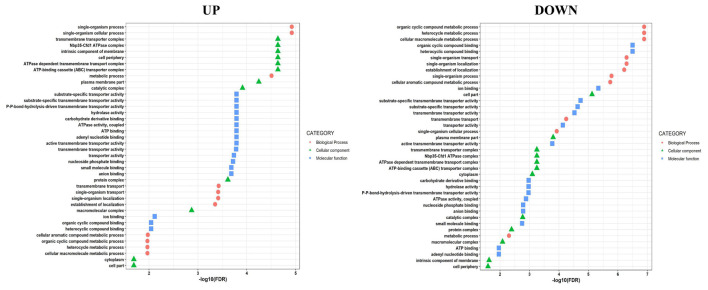
Gene ontology classification in up-regulated and down-regulated genes during PET utilization.

There were 3,769 genes used for GO analysis for identifying the function of DEG. These DEGs are categorized into 76 functional groups and into three categories (i.e., biological process, molecular function, and cellular component) shown in Figure 7 with false discovery rate (FDR) value of less than 0.01 (Supplementary Table 5). The results of GO annotations displayed that ATPase-dependent transmembrane transport complex, ATP-binding cassette (ABC) transporter complex, Nbp35-Cfd1 ATPase complex, and transporter complex were dominant in cellular components, whereas transmembrane transport, single-organism transport, and single-organism localization were dominant in the biological process. The transmembrane transporter activity, ATPase activity, transmembrane movement of substances, primary active transmembrane transporter, and hydrolase activity act on acid anhydrides involved in the molecular function.

### Kyoto Encyclopedia of Genes and Genomes Analysis

**FIGURE 8:**
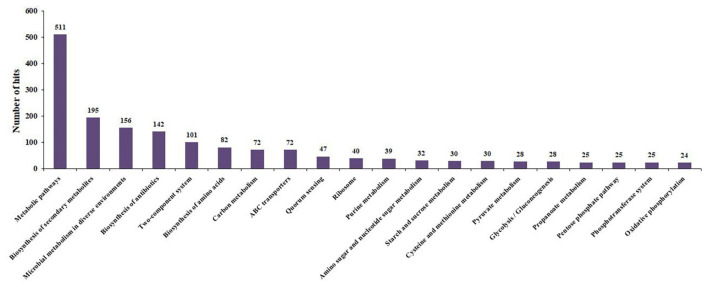
Top 20 KEGG pathways during PET utilization by *Bacillus* species AIIW2.

The biological pathways in the *Bacillus* species AIIW2 grown in PET treatment were mapped to the KEGG database. A total of 1,974 contigs were assigned to 218 KEGG pathways; 511 contigs (25.88%) were involved in metabolic pathways, 156 (7.90%) contigs for microbial metabolism in diverse environments, 72 contigs (3.64%) for ABC transporters, 24 contigs (1.21%) for oxidative phosphorylation, 18 contigs (0.91%) for carbon fixation pathways in prokaryotes, 8 contigs (0.40%) for benzoate degradation, and 5 contigs (0.25%) for biofilm formation (Figure 8 and Supplementary Table 6). Interestingly, most up-regulated pathways were related to an intermediary pathway and quorum sensing such as the Krebs cycle and β-oxidation. However, there are voids observed within the catabolic trails, due to the limited number of enzymes annotation.

### Reverse Transcriptase–Polymerase Chain Reaction Validation of Differentially Expressed Genes

**FIGURE 9:**
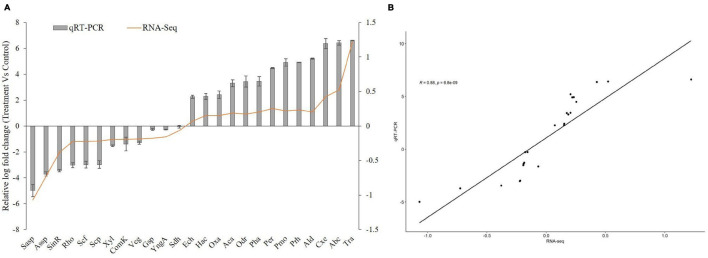
Quantitative RT-PCR of DEGs of *Bacillus* species AIIW2 when grown with PET film compared with culture grown without PET film **(A)**. Correlation analysis between qRT-PCR and RNA-Seq data expression values **(B)**. The *R* and *p* represent the Pearson correlation coefficient and *p* value, respectively.

For the validation of transcriptome expression profile, quantitative RT-PCR analysis (16S rRNA gene used as reference) was performed. The expression of 13 up and 12 down DGEs were validated using RT-PCR. The fold-change expressions were compared with RNA-seq expression profile data (Figure 9 and Supplementary Table 7). RT-PCR showed considerable correlation with transcriptome profile between bacterial culture grown without and with PET, suggesting the consistency and accuracy of the RNA-seq expression analysis.

### Network of Carboxylesterase and Aldehyde Dehydrogenases Gene

**FIGURE 10:**
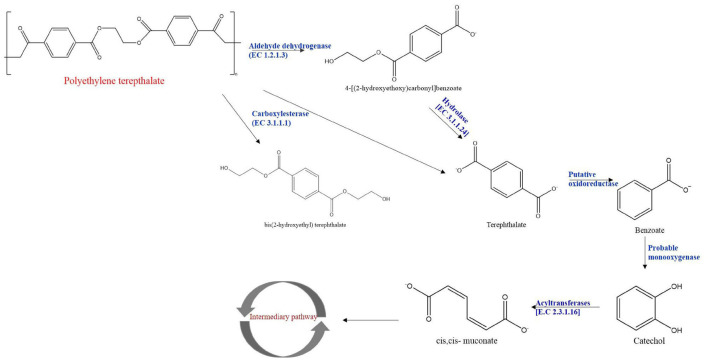
Proposed PET metabolism by *Bacillus* species AIIW2.

**FIGURE 11:**
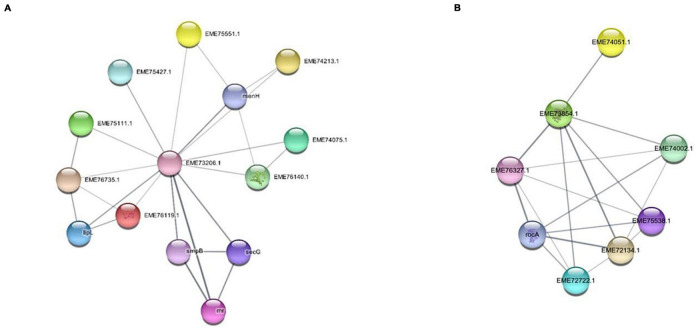
Network analysis of **(A)** carboxylesterase and **(B)** aldehyde dehydrogenase gene. String interaction network displaying the relationship of gene clustered in a module.

The network analysis of carboxylesterase and aldehyde dehydrogenases was performed to understand functional interactions between the expressed proteins by integrating protein–protein interaction with close species based on gene orthology. The protein–protein interaction networks were constructed for carboxylesterases and aldehyde dehydrogenases based on RT- PCR and HPLC data and illustrated pathways for PET biodegradation (Figure 10). The protein–protein interaction networks were constructed for carboxylesterases and aldehyde dehydrogenases based on RT-PCR and HPLC data. The significantly up-regulated genes such as carboxylesterase and aldehyde dehydrogenase that might have a crucial function in PET degradation were used to construct the protein–protein interaction networks (Figure 11). There were 13 nodes and 24 edges detected in carboxylesterase, whereas 7 nodes and 17 edges were detected in aldehyde dehydrogenase, at a confidence (score) cutoff 0.40. Table 1 lists the network interactors and their description, which provided information about the physical and functional associations between proteins and pathways from the database.

**TABLE 1:** Network description of carboxylesterase and aldehyde dehydrogenase of marine *Bacillus* species.

**Gene**	**Interactor**	**Description of interactor**
*Carboxylesterase*	rnr	Ribonuclease R
	smpB	SsrA-binding protein
	secG	Preprotein translocase subunit SecG
	menH	Putative 2-succinyl-6-hydroxy-2,4-cyclohexadiene-1-carboxylate synthase
	lipL	Octanoyl-[GcvH]: protein N-octanoyltransferase
	EME75427.1	Carboxylic ester hydrolase
	EME74075.1	Esterase/lipase
	EME76140.1	Enoyl-[acyl-carrier-protein] reductase (NADH)
	EME75111.1	TPR repeat-containing protein YrrB
	EME75551.1	8-Amino-7-ketopelargonate synthase
	EME74213.1	8-Amino-7-ketopelargonate synthase
	EME76735.1	UPF0354 Uncharacterized protein conserved in bacteria
	EME76119.1	Adapter protein MecA
*Aldehyde dehydrogenase*	EME76327.1	COG1012 NAD-dependent aldehyde dehydrogenases; belongs to the aldehyde dehydrogenase family
	EME75538.1	COG1012 NAD-dependent aldehyde dehydrogenases; belongs to the aldehyde dehydrogenase family
	rocA	L-Glutamate γ-semialdehyde dehydrogenase; COG1012 NAD-dependent aldehyde dehydrogenases; belongs to the aldehyde dehydrogenase family; RocA subfamily
	EME72722.1	COG1012 NAD-dependent aldehyde dehydrogenases; belongs to the aldehyde dehydrogenase family
	EME74002.1	COG1012 NAD-dependent aldehyde dehydrogenases
	EME73854.1	COG1012 NAD-dependent aldehyde dehydrogenases; in the C-terminal section; belongs to the iron-containing alcohol dehydrogenase family
	EME72134.1	COG1012 NAD-dependent aldehyde dehydrogenases; belongs to the aldehyde dehydrogenase family
	EME74051.1	COG1028 dehydrogenases with different specificities (related to short-chain alcohol dehydrogenases)

## Discussion

The present study focused on finding PET-degrading gene in marine *Bacillus* species AIIW2 and illustrating the involved pathway. PET degradation by marine bacterial strain was determined up to 3 months by measuring weight loss and degree of mineralization. The preweighted standard and intact PET film had 1.93% of dry weight reduction after 3 months of incubation with marine *Bacillus* AIIW2 (Figure 1). In a previous study, hydrolase from *T. fusca was* demonstrated to degrade PET-G and PET-B by 50% weight loss in 3 weeks (Mueller, 2006). PET-GF and PET-S degraded approximately 13.5 and 27.0%, respectively, by cutinase from *Saccharomonospora viridis* (Kawai et al., 2014). The CO_2_ evolution test followed up evidence for the biodegradability of a test material. Castro-Aguirre et al. (2017). Kumar et al. (2020) reported 30.52% weight reduction and mineralization of PET into CO_2_ and water after incubating with *Rhodococcus* species grown with terephthalate supplementation for 132 h. The rate of PET mineralization by strain AIIW2 followed up by 128.02 mg CO_2_ g^– 1^ of C evolved cumulatively after 35 days and increased with the incubation period (Figure 2). The carbon dioxide remineralization was 0.051 and 0.046 cm^3^ from cellulose filter paper and Novamont Mater-Bi (constituted 60% starch), respectively, in 5 days of incubation (Mohee et al., 2008). The bacterial colonization on the PET film is an essential prerequisite in biodegradation. The SEM images of bacteria-treated PET film support the efficient attachment and degradation of the surface compared with control film (Figure 3). Koshti et al. (2018) suggested that the bacterial colonization on the PET surface was an initiation of bacterial action for the degradation activity. The surface contact angle reduced from 77.3° to 55.8° of the PET film after 90 days of bacterial incubation. The reduction in water contact angle of PET surface represents a decreased hydrophobicity and increased hydrophilicity after 90 days of incubation with *Bacillus* species AIIW2 (Figure 4), which is in corroboration with the observations of Radhakumary et al. (2005) and Arutchelvi et al. (2008). The contact angle of corona discharge treatment and UV-treated polyethylene was found to be reduced significantly up to 54.6% in corona discharged film and 24.56% in UV-treated film after incubation with fungal consortium (Matsunaga and Whitney, 2000). The contact angle of chitosan-blended polyethylene matrix was found to be decreased with an increase in chitosan percentage in structure due to the hydrophilic property of chitosan in comparison to unplasticized polyethylene film; moreover, the cross-linking of palm oil also added hydrophilicity in the LDPE structure incubated with *Aspergillus niger* (SunilKumar et al., 2012). The water contact angle of PET film treated with *Bacillus subtilis* was found to be reduced from 68.2° to 62.6° (Ribitsch et al., 2011).

The formation of KCBI and ECBI and reduction in IDBI and VBI were observed by FTIR spectra that confirmed the structural transformation of PET (Figure 5). The formation of acids (1,715 cm^– 1^) and ketones (1,740 cm^– 1^) and disappearance of double bonds (1,640 and 908 cm^– 1^) were analyzed using the FTIR to elucidate the PET biodegradation process. Notably, the appearance of hydrolyzable carbonyl functional group in the polymer structure will provide the site for hydrolytic enzyme action for biotransformation. The increase of KCBI and ECBI showed oxidoreductive enzyme activity on PET film incubated with strain AIIW2. The reduction in VBI and IDBI indicated the deformation and reduction in internal structure, whereas there was negligible variation in the bond index in PET film incubated without bacteria. The presence of carbonyls bonds as a product of degradation suggests the presence of oxidoreductive enzymes (Harshvardhan and Jha, 2013). The formation of double bonds or esters in the polymer chain may be due to the Norrish type II reaction proposed earlier (Albertsson et al., 1987). Janczak et al. (2018) reported structural changes in PET film after incubation with rhizospheric microbe in carbonyl bond indexes. The carbonyl bond indexes, that is, KCBI, ECBI VBI, and IDBI of HDPE, were increased when incubated with *Arthrobacter* species; however, whereas in the incubation with *Pseudomonas* species KCBI, ECBI, and VBI increased, IDBI decreased compared with the control (Balasubramanian et al., 2010).

The heat map represents that the genes involved in carbon metabolism and cell transport system were up-regulated in cells growing with PET, whereas sporulation genes were expressed in the absence of PET. This observation indicates the nodes of PET assimilation via cellular carbon metabolism by strain AIIW2 (Figure 6). A similar pattern was also observed in GO; that is, biological processes, molecular functions, and cellular components genes were up-regulated while growing with PET film (Figure 7). In a study on polyethylene degradation by *Rhodococcus ruber* strain, fatty acid degradation, alkane degradation, and β-oxidation pathways were the most up-regulated pathways (Gravouil et al., 2017).

In this study, the KEGG analysis showed that supplementation of PET induces the bacterial assimilation pathway for its utilization; indeed, the *Bacillus* species AIIW2 grows when PET is provided as a carbon source (Figure 8). The top 13 up-regulated genes were identified as being differentially expressed in PET treated with bacterial cultures. The Ct averages and standard error were calculated to find the log-fold expression of the genes in the treated and control samples. The RT-PCR results also displayed the highest relative fold change in carboxylesterase (EC 3.1.1.1), ABC transporter, and transporter genes predicted to have 6.37, 6.48, and 6.61, respectively, in PET treated in comparison to control *Bacillus* species AIIW2 grown in the absence of PET film (Figure 9). The secretion of hydrolytic enzyme that hydrolyzes the PET structure results in carbonyl bond index and reduction in internal as well as vinyl bonds (Albertsson et al., 1987; Balasubramanian et al., 2010). Further, the transport system helps the subsequently hydrolyzed oligomers for cellular assimilation (Gravouil et al., 2017). The release hydrolysis product of PET during incubation with *Bacillus* species AIIW2 was quantified by reverse-phase HPLC. The presence of BHET, MHET, and TPA as a degradation product aldehyde dehydrogenase and esterase activity involved in the initial degradation step, although in combination with expression profiling (RNA-seq and RT- PCR), suggest the same. The concentrations of released BHET, MHET, and TPA were found to be 0.34, 14.01, and 107.06 mM, respectively, in 30 days of incubation by HPLC analysis. The TPA was found to be a major hydrolytic product suggesting the PET hydrolyzed into BHET, MHET, and TPA, where TPA would further hydrolyze from MHET by carboxylesterase and hydrolase (Barth et al., 2015; Yoshida et al., 2016). Yoshida et al. (2016) proposed the degradation of PET by *Ideonella sakaiensis* through hydrolysis of PET into MHET and TPA extracellularly and further transported into the cell to catabolism. In a study, TPA was released higher than MHET as a resulting product of novel esterase from *T. halotolerans*, where amorphous PET fiber was hydrolyzed into BHET (Ribitsch et al., 2012). The cutinase from *Fusarium solani* hydrolyzed PET into BHET, MHET, and TPA, although MHET and TPA ratios were higher (Vertommen et al., 2005). The degradation pathway was elucidated based on changes in the functional groups and HPLC determination. Carboxylesterase is likely to hydrolyze PET into BHET and TPA, whereas MHET by aldehyde dehydrogenase activity (Figure 10).

In a study, a network analysis of degrading protein obtained from the genome of *Sphingopyxis* strains deciphers interaction with the core content (Verma et al., 2020). Although carboxylesterase and aldehyde dehydrogenase genes from RNA-seq data were mapped for interaction with a relative set of hydrolytic proteins, the 13 genes of carboxylesterase were mapped, and interactors of catabolic and central metabolism were identified with both sets of genes. This determines the distant placement of proteins for growth and metabolism while growing with an unusual substrate such as PET. Interactome network analysis provided the relationship of carboxylesterase genes (EME73206.1) with the interactors, such as rnr, smpB, secG, menH, lipL, EME75427.1, EME74075.1, EME76140.1, EME75111.1, EME75551.1, EME74213.1, EME76735.1, and EME76119.1 (Barth et al., 2016), and seven genes of aldehyde dehydrogenase (EME73854.1) from the iron-containing alcohol dehydrogenase family were mapped, and their interactors were EME76327.1, EME75538.1, EME72722.1, EME74002.1, EME72134, rocA L-glutamate γ-semialdehyde dehydrogenase belonging to the aldehyde dehydrogenase family, EME74051.1 dehydrogenase with different specificities (short-chain alcohol dehydrogenases) (Reid and Fewson, 1994). The network analysis of carboxylesterase showed that interactions were present between proteins from essential cellular metabolism, such as ribonuclease R, SsrA-binding protein, preprotein translocase subunit SecG, putative 2-succinyl-6-hydroxy-2,4-cyclohexadiene-1-carboxylate synthase, N-octanoyl transferase, carboxylic ester hydrolase, esterase/lipase, enoyl-[acyl-carrier-protein] reductase (NADH), TPR repeat-containing protein YrrB, 8-amino-7-ketopelargonate synthase, 8-amino-7-ketopelargonate synthase, UPF0354 uncharacterized protein conserved in bacteria, and adapter protein MecA. Similarly, aldehyde dehydrogenase interactors were NAD and short-chain alcohol dehydrogenases (Figure 11). The identification of interactions between genes and between proteins established elucidation of the functions of genes of interest or for a better understanding of a PET degradation process.

## Conclusion

PET is the most common contributor to solid wastes in the environment. A growing body of literature supports microbial degradation of PET polymer; however, it cannot resolve how degradation occurs. Marine bacterium *Bacillus* species AIIW2 had the ability to degrade PET structure and remineralize into CO_2_. The comparative transcriptome profile of strain AIIW2 suggested that hydrolytic enzymes were playing a key role during the utilization of PET film by the bacteria. The degradation product and predicted pathway have suggested the role of aldehyde dehydrogenase and carboxylesterase in PET hydrolysis. The genes traced to degrade PET were confirmed by detecting the hydrolytic product of PET and carboxylesterase gene found to be a key enzyme in the degradation process and selected for cloning. The degradation pathway had been elucidated based on changes in the functional group and HPLC determination. We provide transcriptome-based insight into the biodegradation of PET films by marine bacteria *Bacillus* species AIIW2, although the gene annotated during the degradation process could help to develop an engineered microbial system for improved degradation of PET. The study will establish an understanding of plastic biodegradation through microbial resources as eco-friendly for reducing pollution. Altogether, this study provides a map involved during the PET degradation by marine bacterial strain.

## Data Availability Statement

The datasets presented in this study can be found in online repositories. The names of the repository/repositories and accession number(s) can be found below: GenBank, MZ322848.

## Author Contributions

AK performed the experiments and drafting the manuscript. DC and BJ contributed to planning the experiment and manuscript preparation. NB and SB performed the bioinformatics analysis. All authors contributed to the article and approved the submitted version.

## Conflict of Interest

The authors declare that the research was conducted in the absence of any commercial or financial relationships that could be construed as a potential conflict of interest.

## Publisher’s Note

All claims expressed in this article are solely those of the authors and do not necessarily represent those of their affiliated organizations, or those of the publisher, the editors and the reviewers. Any product that may be evaluated in this article, or claim that may be made by its manufacturer, is not guaranteed or endorsed by the publisher.

## Footnotes

1
http://www.genome.jp/tools/kaas/

2
https://www.genome.jp/kegg/mapper.html

3
http://string-db.org/

## Supplementary Material

Supplementary Figure 1 Water contact angle images on PET film surface after incubating with *Bacillus* species AIIW2.

Supplementary Table 1 Quantitative RT-PCR primers sequence.

Supplementary Table 2 Description of high-quality read. ^∗^ C1_R1, C1_R2; C2_R1, C2_R2; C3_R1, C3_R2—paired reads of control samples in triplicates. ^∗^ T1 R1, T1 R2; T2 R1, T2 R2, T3 R1, T3 R2—paired reads of PET treated samples in triplicates.

Supplementary Table 3 A detailed description of filtered reads for mRNA-seq.

Supplementary Table 4 Up- and down-regulated genes in *Bacillus* AIIW2 growing with PET film.

Supplementary Table 5 Genes and their annotations of *Bacillus* AIIW2 growing with and without PET film mRNA-seq profile.

Supplementary Table 6 GO annotation and functional categorization of DEGs in *Bacillus* AIIW2 growing with PET film.

Supplementary Table 7 RT-PCR and RNA-seq expression correlation data.
