# Supplementary figures and images for: Transcriptome-Guided Insights Into Plastic Degradation by the Marine Bacterium
Source: Front Microbiol. 2021 Sep 27;12:751571. doi: 10.3389/fmicb.2021.751571 (PMC8503683; doi:10.3389/fmicb.2021.751571)

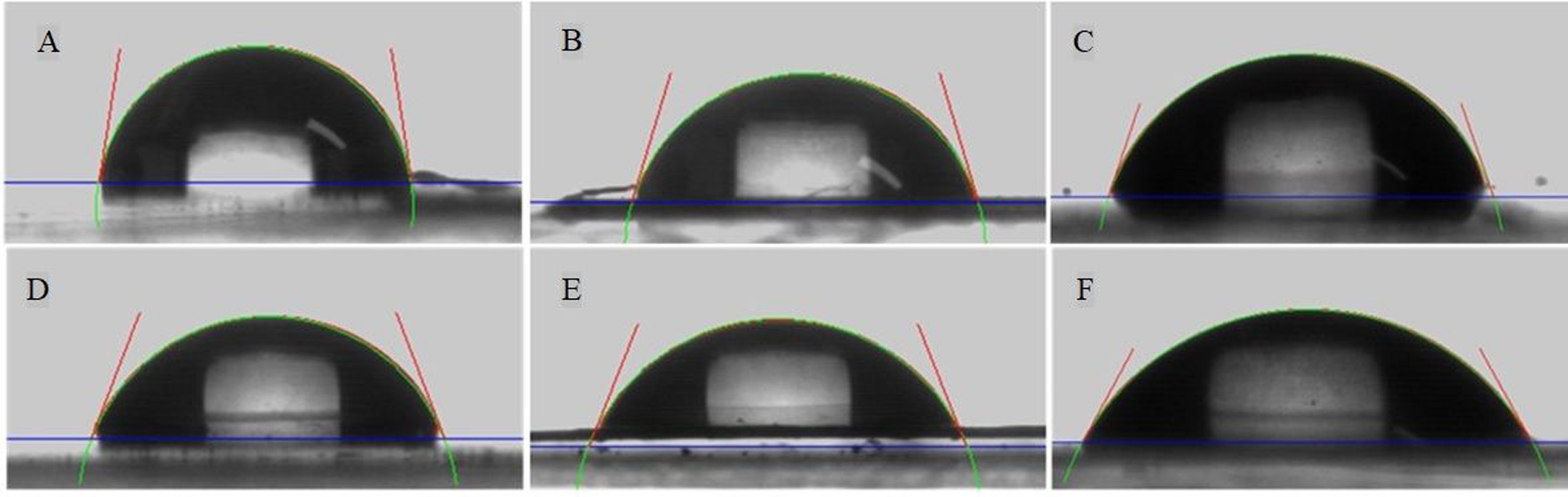

Supplement: Supplementary Figure 1 — Water contact angle images on PET film surface after incubating with Bacillus species AIIW2. [file Image_1.JPEG]
